# Prevalence of exclusive breastfeeding practice in the first six months of life and its determinants in Iran: a systematic review and meta-analysis

**DOI:** 10.1186/s12887-019-1776-0

**Published:** 2019-10-27

**Authors:** Meysam Behzadifar, Mandana Saki, Masoud Behzadifar, Mahnaz Mardani, Fatemeh Yari, Farzad Ebrahimzadeh, Hadis Majidi Mehr, Shadi Abdi Bastami, Nicola Luigi Bragazzi

**Affiliations:** 1 Social Determinants of Health Research Center, Lorestan University of Medical Sciences, Khorramabad, Iran; 2 Health Management and Economics Research Center, Iran University of Medical Sciences, Tehran, Iran; 3 Nutritional Health Research Center, Lorestan University of Medical Sciences, Khorramabad, Iran; 4 Department of Midwifery, Faculty of Nursing and Midwifery, Lorestan University of Medical Sciences, Khorrmabad, Iran; 5 Department of Biostatistics, School of Health and Nutrition, Lorestan University of Medical Sciences, Khorramabad, Iran; 6 Department of Public Health, Faculty of Health and Nutrition, Lorestan University of Medical Sciences, Khorramabad, Iran; 7 School of Public Health, Department of Health Sciences (DISSAL), University of Genoa, Genoa, Italy

**Keywords:** Prevalence, Exclusive breastfeeding, Iran, Systematic review, Meta-analysis

## Abstract

**Background:**

Exclusive breastfeeding (EBF) in the first 6 months of life is the best and most complete option for an infant, in that supplies the vitamins and minerals the baby needs. Several studies in Iran have been conducted concerning the prevalence of EBF. The aim of this study was to determine the prevalence of EBF in the first 6 months of life and associated factors in Iran synthesizing published studies.

**Methods:**

We searched PubMed/MEDLINE, Embase, Scopus, ISI/Web of Science, the Cochrane Library, Directory of Open Access Journals Directory (DOAJ) and Google Scholar as well as Iranian databases (Barakathns, MagIran and the Scientific Information Database or SID) up to November 2018. The Newcastle-Ottawa Scale was used to assess the quality of studies. Analyses were performed by pooling together studies using DerSimonian-Laird random-effects model with 95% confidence interval. To test for heterogeneity, I^2^ test was used. The Egger’s regression test and funnel plot were used to evaluate the publication bias. The strength of EBF determinants was assessed computing the Odds-ratios (OR) using the Mantel–Haenszel method.

**Results:**

In the initial search 725 records were found. Finally, 32 studies were selected based on inclusion/exclusion criteria. The sample size of studies varied between 50 and 63,071 subjects. The overall prevalence of EBF in Iran was 53% (CI 95%; 44–62). The OR for breastfeeding education received before pregnancy was 1.13 (0.94–1.36), for mother’s job 1.01 (0.81–1.27), for education level 1.12 (0.89–1.42), for type of delivery 1.16 (0.98–1.37), and for gender of child 1.03 (0.83–1.28).

**Conclusion:**

In Iran health policy- and decision-makers should try to take interventions that encourage mothers to use their milk to breastfeed the infants.

## Background

Exclusive breastfeeding (EBF) in the first 6 months of life is known to be the most complete nutrient for a newborn, in that it provides all the energy, vitamins and minerals the baby needs [1, 2]. As the World Health Organization (WHO), the American Academy of Pediatrics (AAP) and the United Nations Children’s Fund (UNICEF) emphasize, it is important for an infant to receive only breast milk up to the first 6 months of age, whereas, after the first 6 months, breast milk can be given in addition to other foods [2–4].

However, despite its importance and its clinical implications, both in developed and developing countries, the full implementation of EBF practice encounters some obstacles and barriers. As such, health policy- and decision-makers should pay particular attention to this issue, making their efforts to design ad hoc programs for EBF promotion [5]. The best cost-effective intervention to reduce mortality in countries is, indeed, to increase compliance to EBF practice [6].

EBF, both in the short and long term, has many benefits for the infant and the mother, which can curb the costs of infant care and nutrition, reduce the occurrence of several infectious diseases [7]. EBF is also effective in mitigating the burden of non-communicable diseases such as diabetes, asthma and cardiovascular disease in later years [8–10]. Despite the vast benefits of EBF, only half of infants under 1 month and about 30% of infants from 1 to 5 months are breastfed [11]. In studies conducted for estimating EBF prevalence and understanding its determinants, different factors have been individuated, including mother’s awareness and positive attitude towards EBF, her socioeconomic and employment status, setting (urban versus rural areas), type of delivery, and weight of the baby at the time of birth [12–14].

The prevalence of EBF in the first 6 months of life in different countries has been explored. In a study conducted in a developing country (India), the prevalence was reported to be 34% [15]. Also, the prevalence rates of EBF in Turkey (38.9%), in Tanzania (20.7%), in Syria (12.9%), and in Egypt (9.7%) were reported [16–19]. Concerning prevalence of EBF in developed countries, in a study conducted in the United States, the rate was 16.8% [20]. The prevalence rates of EBF in other contests, including Spain (31.4%), Canada (13.8%) and Italy (5.5%), were also documented [21–23].

Various studies have been conducted in Iran too, in order to evaluate the prevalence of EBF. Therefore, the aim of this study was to determine the prevalence of EBF and to study its determinants in Iran, summarizing the existing available investigations. The findings of this study can be helpful for health policy- and decision-makers, planners, mothers, doctors, and all the other stakeholders in the field of healthcare in selecting effective interventions for the promotion of EBF practice.

## Methods

The findings of this study were reported according to the “Preferred Reporting Items for Systematic Reviews and Meta-Analyzes” (PRISMA) Guidelines [24].

### Search strategy

We searched different scholarly electronic databases, namely PubMed/MEDLINE, Embase, Scopus, ISI/Web of Science, the Cochrane Library, Directory of Open Access Journals Directory (DOAJ) and Google Scholar as well as Iranian databases (Barakathns, MagIran and the Scientific Information Database, SID) up to November 2018. The search terms used were: (“exclusive breastfeeding” OR “breastfeeding” OR “breast-feeding” OR “breastfeeding patterns” OR “breastfeeding practices” OR “breastfeeding status” OR “feeding status”) AND (“frequency” OR “epidemiology” OR “prevalence” OR “patterns” OR “assessment” OR “investigation”) AND “Iran”. The reference list of included studies was also scanned in order to obtain relevant additional studies. A search strategy adapted to PubMed/MEDLINE, Scopus, ISI/Web of Science and Embase is reported in appendix (Additional file 1).

### Inclusion criteria

Inclusion criteria were: 1) studies in which mothers used their milk to feed their infants up to 6 months of age; 2) studies reporting the prevalence of EBF in the first 6 months of life; 3) studies in which babies were aged more than 6 months; 4) studies whose data were sufficient to calculate the prevalence; 5) studies published in peer-reviewed journals; and 6) studies written either in Persian or English.

### Exclusion criteria

Exclusion criteria were the following: 1) studies designed as case-series, case-reports, randomized clinical trials, or interventional investigations; 2) studies whose data were inadequate or insufficient to estimate the prevalence of EBF; and 3) studies unavailable in full-text.

### Outcome measurement

The outcomes of interest of this study included: 1) the prevalence of EBF practice in the first 6 months of life in Iranian children; and 2) the determinants of EBF practice.

### Data extraction

We extracted the following data from the studies included in the present systematic review and meta-analysis: first author, year of publication, location, sample size, number of breastfed children, mothers’ age, reported prevalence, determinants of EBF, study design, and language of study.

### Quality assessment (risk of bias)

The Newcastle-Ottawa Scale (NOS) was used to assess the quality of studies. This tool consists of three major sections, concerning the methodological quality, the comparability and the outcomes and statistical analysis of each included study. Two authors independently critically appraised the quality of each original study using the NOS tool. Disagreements between the two authors were resolved by consensus. According to the stars assigned to each part, the studies with at least 5 stars out of 10 were considered of good quality [25].

To extract relevant data and to evaluate the quality of the studies, two authors independently performed these steps. In case of disagreement, consensus was reached through discussion. Kappa statistics was used to assess the agreement between the two authors. Kappa coefficient was 0.93 for data extraction and 0.81 for evaluation of study quality.

### Statistical analysis

All analyses were conducted using Stata Version 12 (Stata Corp, College Station, TX, USA) utilizing the “metaprop” command [26]. Overall pooled estimates with inverse-variance weights analyses were performed by logistic-normal random-effects model using DerSimonian-Laird approach with 95% confidence interval (CI) [27]. In order to stabilize the variance the double arcsine transformation method according to Freeman and Tukey was used [28].

To test for heterogeneity, I^2^ test was utilized [29]. Subgroup analyses were conducted based on the sample size, the geographical area, and other variables such as education concerning EBF received before/during pregnancy, mother’s job, education level, type of delivery, gender of child, birth weight, mother’s diseases/co-morbidities and location of delivery. The Egger’s linear regression test and the funnel plot were used to evaluate the publication bias [30]. To determine possible sources of heterogeneity, meta-regressions were carried out based on the year and the sample size of the studies. Also, sensitivity analysis was performed to check the stability of results. To assess the strength of the different determinants of EBF practice, odds Ratio (OR) with its 95% CI was calculated using the Mantel–Haenszel method.

In all the statistical analyses, figures with a *p*-value less than 0.05 were considered statistically significant.

## Results

### Findings of the search strategy

**Fig. 1:**
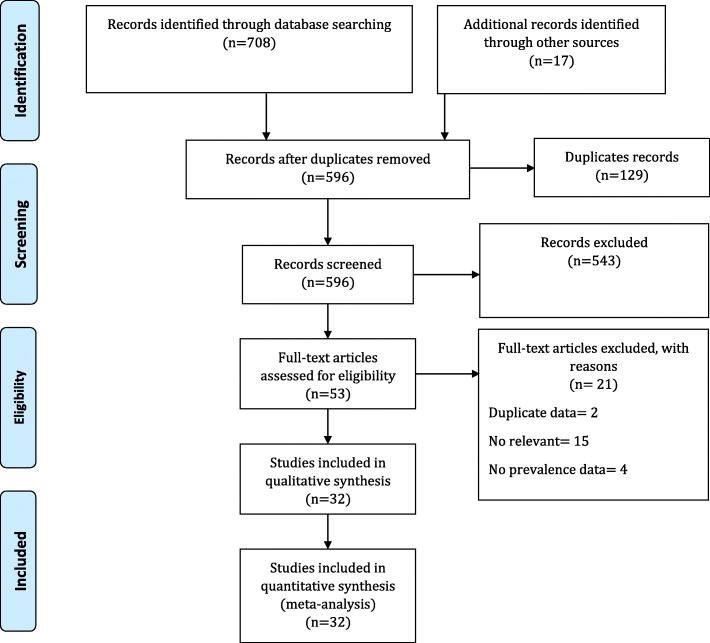
Flowchart illustrating the process of search and selection of studies included in the present systematic review and meta-analysis

In the initial search, 725 records were found. After removing duplicates, the title of 596 records was checked and 543 records were deleted. The abstract of 53 studies was then reviewed and, finally, the full text of 32 studies was selected based on inclusion/exclusion criteria. Figure 1 shows the process of the search and selection of studies.

### Main characteristics of included studies

Selected studies were conducted between 2003 and 2015 [31–62]. The sample size of studies varied between 50 and 63,071 subjects. The main characteristics of retained studies are presented in Table 1.

**Table 1:** The characteristics of studies included

First author	References	Year of publication	Region	Age of mothers (Mean ± SD)	Age of baby (month)	Sample size	Study design	Quality rating of the studies (Stars) (risk of bias)
Imani	31	2003	Zahedan	NA	6–24	253	Cross-sectional	6 stars
Hajian-Tilaki	32	2005	Babol	NA	6	600	Cross-sectional	8 stars
khabazkhoob	33	2008	Mashhad	NA	7–12	1267	Cross-sectional	7 stars
Koosha	34	2008	Zanjan	NA	12	50	Cross-sectional	6 stars
Mohsenzadeh	35	2008	Khorramabad	NA	12	340	Cross-sectional	8 stars
Mohammad Beygi	36	2009	Arak	NA	6–12	352	Cross-sectional	9 stars
Olang	37	2009	30 provinces	NA	< 24	63,071	Retrospective	8 stars
Roudbari	38	2009	Zahedan	25.5 ± 6.2	12	450	Cross-sectional	7 stars
Almasi	39	2010	Kashan	NA	6	391	Cross-sectional	8 stars
Vafaee	40	2010	Mashhad	NA	12	1450	Cross-sectional	9 stars
Hamidi	41	2011	Charmahalva Bakhtiari	29.25 ± 5.5	< 12	411	Descriptive-analytical	7 stars
Mehrparvar	42	2011	Kerman	NA	< 12	320	Cross-sectional	7 stars
Naserpoor	43	2011	Omidieh	27.5 ± 5.5	6–18	400	Descriptive-analytical	8 stars
Rahmatnejad	44	2011	Tehran	NA	12	331	Cross-sectional	8 stars
Torabi	45	2011	Jahrom	28.1 ± 5.36	18–24	435	Cross-sectional	7 stars
Veghari	46	2011	Golestan	NA	6–60	2520	Cross-sectional	8 stars
Yaghini	47	2011	Isfahan	NA	12	656	Descriptive-analytical	6 stars
Kermani	48	2012	Tehran	NA	6	110	Cross-sectional	9 stars
Mirahmadizadeh	49	2012	Shiraz	NA	6–12	751	Historical cohort	8 stars
Morowatisharifabad	50	2012	Ardakan	NA	6–12	413	Cross-sectional	8 stars
Ziaie	51	2012	Rasht	30.93 ± 4.801	< 12	263	Descriptive-analytical	7 stars
Charkazi	52	2013	Isfahan	27.79 + 4.7	6–24	406	Cross-sectional	8 stars
Kamali	53	2013	Tehran	28.9 ± 4.6	12–24	300	Cross-sectional	6 stars
Khamnian	54	2013	East Azerbaijan	NA	12	750	Cross-sectional	8 stars
Saki	55	2013	Shiraz	NA	12	287	Prospective follow-up	7 stars
Abdollahi	56	2014	Sari	27.99 ± 4.7	< 12	400	Cross-sectional	9 stars
Aghababaii	57	2014	Hamadan	26.7 ± 4.8	12	1200	Cross-sectional	8 stars
Dalili	58	2014	Tehran	NA	6	175	Cross-sectional	7 stars
Ghanbarnejad	59	2014	Bandar Abbas	25.7 ± 5.6	6	800	Cross-sectional	7 stars
Noughabi	60	2014	Tehran	NA	6–24	538	Cross-sectional	8 stars
Ranjbaran	61	2014	Shazand	NA	6	283	Cross-sectional	8 stars
Roostaee	62	2015	Zahedan	NA	12	523	Cross-sectional	9 stars

### Findings of the quality assessment

According to the NOS tool, the quality assessment showed that 4 studies were scored 6 stars, 9 studies 7 stars, 14 studies 8 stars, and 5 studies 9 stars. No study was excluded after rating because the study quality was always above 5 stars. Result of assessment of risk of bias for each study are reported in Table 1.

### Findings of the meta-analysis

**Fig. 2:**
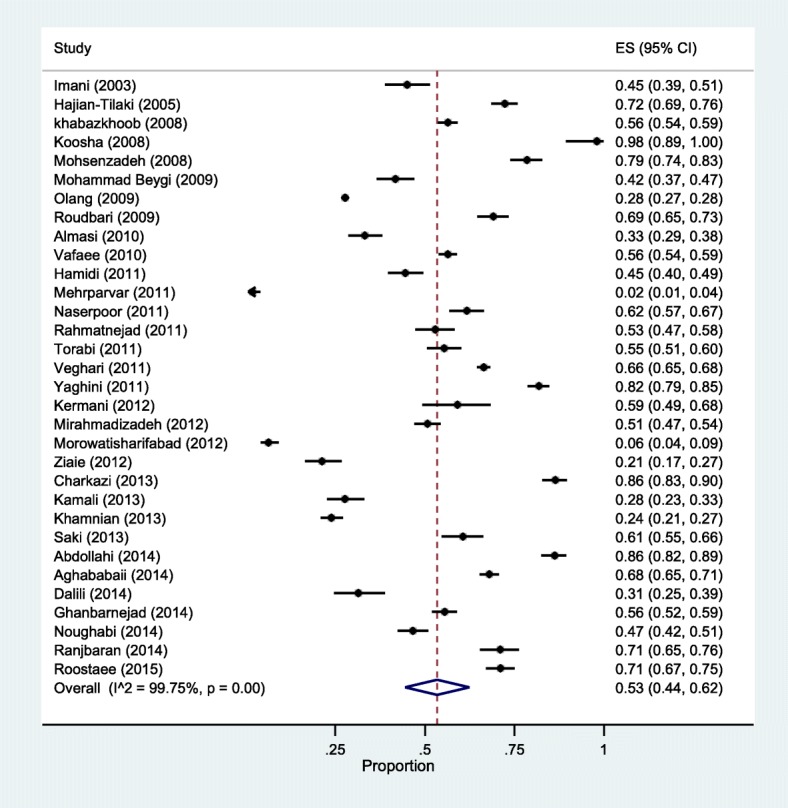
The overall prevalence of EBF in Iran

Based on DerSimonian-Laird model, EBF prevalence in Iran was computed to be 53% (CI 95%; 44–62) (Fig. 2). Heterogeneity resulted statistically high, I^2^ = 99.7%, *P* = 0.000. Sensitivity analysis also showed that the results did not change before and after the analysis and confirmed the stability of the results.

### Findings of the subgroup analysis

Table 2 shows the results of the subgroup analysis. According to the geographical area of the study, the highest prevalence of EBF was observed in the north (62% versus 61% in the west, 60% in the east, 48% in the south, and 47% in the center of Iran). In terms of sample size, in studies with a sample size comprising more than 500 subjects, the prevalence was 56%, and 52% in studies with less than or equal to 500 individuals. The prevalence of EBF in mothers who had been educated before and during pregnancy was 55% and 50%, respectively. The prevalence of EBF in unemployed and employed mothers was 58% and 55%, respectively. In terms of educational level, the prevalence of EBF in mothers without and with higher education was 58% and 56%, respectively. The prevalence of EBF in mothers who delivered vaginally was 58% and 49% in mothers who underwent cesarean section. The prevalence of EBF stratified according to the gender of baby was 60% and 50% in case of female and male, respectively. The prevalence of EBF in infants weighing less than 2500 g was 62% while was 60% in infants weighing more than 2500 g. In mothers with diabetes, kidney, and cardiovascular disease, the prevalence of EBF was 44%, while it was 50% in healthy mothers. The prevalence of EBF in mothers giving birth at government hospitals was 69% and 51% in mothers who delivered their babies in private hospitals.

**Table 2:** The results of subgroup-analyses

Variables	Number of studies	Prevalence (95% CI)	Heterogeneity	
			I^2^	*P*-Value
Geographical region				
North	4	62% (42–81)	99.4%	0.000
South	6	48% (20–75)	99.7%	0.000
West	7	61% (41–81)	99.5%	0.000
East	5	60% (52–67)	95.3%	0.000
Center	9	47% (24–70)	99.7%	0.000
Sample size				
≤ 500	20	52% (35–68)	99.7%	0.000
> 500	12	56% (42–70)	99.8%	0.000
Education before pregnancy				
Yes	6	55% (39–71)	98.4%	0.000
No	6	50% (35–64)	88.5%	0.000
Maternal employment				
Unemployed	6	58% (46–69)	95.8%	0.000
Employed	6	55% (37–73)	92.9%	0.000
Education level				0.000
Under dioploma	6	58% (51–64)	52.4%	0.000
Upper dipoloma	6	56% (42–69)	97.3%	0.000
Type of delivery				0.000
Vagina	5	58% (43–74)	97.1%	0.000
Cesarian	5	49% (34–64)	95.7%	0.000
Gender of child				0.000
Girl	4	60% (40–80)	97.6%	0.000
Boy	4	59% (41–78)	96.8%	0.000
Birth weight				0.000
Under 2500 g	3	62% (54–70)	0%	0.000
Upper 2500 g	3	60% (40–80)	97.8%	0.000
Mother with history of diseases (Diabetes, hypertension, …)				0.000
Yes	2	44% (20–68)	86.6%	0.000
No	2	50% (24–76)	85.7%	0.000
Location of delivery				0.000
Govermental	2	69% (45–93)	97.5%	0.000
Private	2	51% (34–71)	98.6%	0.000

### Determinants of exclusive breastfeeding in Iran

Association between some variables and prevalence of EBF was considered in Table 3. In this table, the strengths of the determinants of EBF practice based on the OR computed according to the Mantel–Haenszel method are reported. More in detail, the OR for breastfeeding education received before pregnancy was 1.13 (0.94–1.36), for mother’s job 1.01 (0.81–1.27), for education level 1.12 (0.89–1.42), for type of delivery 1.16 (0.98–1.37), and for gender of child 1.03 (0.83–1.28). All of these predictors were not statistically significant, even though suggestive of a trend.

**Table 3:** Odds-ratios for the different determinants of EBF practice

Variables	Number of studies	Odds ratio (95% CI)	I^2^	*P*-Value
Education concerning breastfeeding received before pregnancy	6	1.13 (0.94–1.36)	0%	0.93
Mother’s job	6	1.01 (0.81–1.27)	0%	0.60
Education level	6	1.12 (0.89–1.42)	25.2%	0.24
Type of delivery	5	1.16 (0.98–1.37)	21.1%	0.28
Gender of child	4	1.03 (0.83–1.28)	39.1%	0.17
Birth weight	3	1.15 (0.86–1.55)	0%	0.43
Mother with history of diseases (Diabetes, hypertension, …)	2	0.94 (0.58–1.52)	0%	0.96
Location of delivery	2	1.32 (0.56–3.11)	78.8%	0.03

### Findings of the meta-regressions

Meta-regressions were performed based on the year of publication and the sample size; the results are shown in Table 4. Based on the year of publication (*P* = 0.61) and the sample size (*P* = 0.26) of included studies, EBF exhibits a decreasing trend throughout the time, even though not statistically significant.

**Table 4:** The results of meta-regressions

Variables	Coefficient	SE	T	*P*- Value	CI 95% Lower	CI 95% Upper
Year of publication	−0.00	0.01	−0.51	0.61	−0.03	0.02
Sample size	−4.41	3.8	−1.15	0.26	−0.00	3.44
Constant	16.48	31.46	0.52	0.60	−47.86	80.82

### Publication bias

**Fig. 3:**
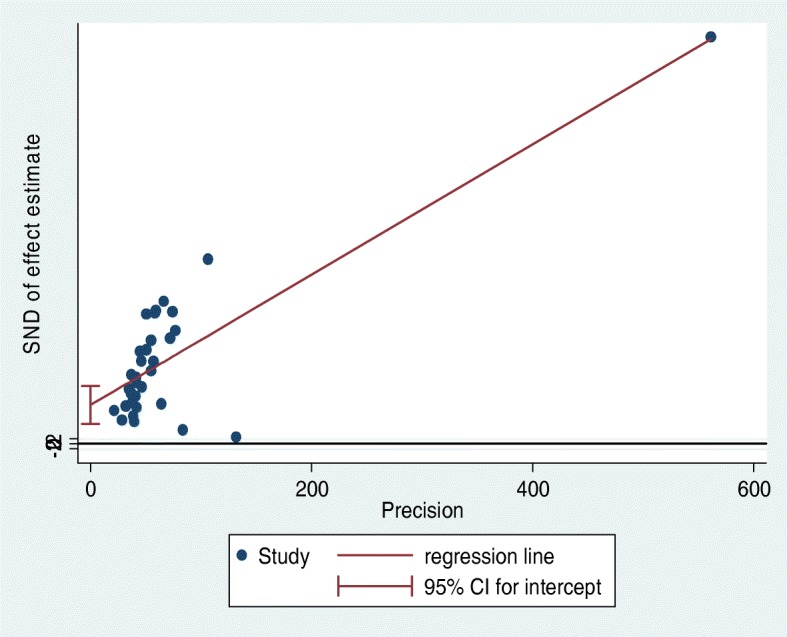
The results of the publication bias analysis based on the Egger’s linear regression test and the visual inspection of the funnel plot

Using the Egger’s linear regression test, the publication bias of included studies was investigated, and resulted not statistically significant (*P* = 0.27), as pictorially shown in Fig. 3.

## Discussion

The aim of this study was to investigate the prevalence of EBF practice in the first 6 months of life in Iran synthesizing available published studies.

### Determinants of exclusive breastfeeding in Iran

In this systematic review and meta-analysis, based on the data from included studies, we examined the determinants of EBF. Training received before and during pregnancy can have an impact on the three dimensions of knowledge, attitudes and behavior of the mothers and encourage them to practice EBF [63]. The findings of this study showed, indeed, that EBF in mothers who received training was higher than in untrained mothers.

Pregnant women and their spouses should be carefully informed about infant birth and breastfeeding, an integral part of prenatal care. Other members of the family who can support breastfeeding can be trained too [64]. Training can be done at health centers and clinics. Hospitals and other institutions can also provide training for pregnant women and their partners. Other health system staff, such as pediatricians, nurses and midwives, play an important role, as well as mother-to-mother education groups and other organizations [65].

Maternal occupation was one of the factors contributing to an increase in the prevalence of EBF. The findings of this study showed that the prevalence of EBF in Iranian housewife mothers was higher than that of employed mothers. This finding is consistent with the results of studies carried out in Ethiopia [66], Saudi Arabia [67], Canada [22] and Jordan [68].

Mothers who work suffer from lack of time, and fatigue, and may find difficulties in breastfeeding [69]. Employment regulations play an important role in promoting EBF practice, by giving mothers more time to breastfeed their babies [70]. On the other hand, it seems that postpartum leave is more likely to lead to an increase in EBF. As such, postpartum mothers need more support from their employers [71].

At present, women in Iran can use 6 months of maternity leave, and their husbands can use 2 weeks. This law is better enforced in governmental organizations but not in many nongovernmental organizations. Although policy- and decision-makers are making a lot of efforts to increase the application of this law, they still have problems such as lack of support from insurance organizations, from employers, and lack of sufficient funding [72].

The findings of this study showed that the prevalence of EBF in less literate women is higher than that of women with university education, which is consistent with findings from studies conducted in Bangladesh [5], United Arab Emirates [73] and Ethiopia [74]. Mothers with lower education appear to be more interested in EBF education. Maternal education is recognized as an important social component for promotion and health-care of children [75]. In a systematic review carried out in high-income countries, results showed that interventions designed and implemented for educational purposes significantly increased the practice of EBF [76].

In our study, the findings showed that the prevalence of EBF in women who gave birth vaginally was higher than that of mothers who had cesarean section, which is consistent with the results of studies performed in Ecuador [77], Saudi Arabia [78], and Jordan [68]. The results of a meta-analysis of 53 studies showed that EBF rates were lower in women with cesarean delivery than in women with vaginal delivery [79]. Health-care providers should increase the awareness of women concerning delivery. Cesarean section is, indeed, associated with special surgical procedures and the use of local anesthesia. It is characterized by a high probability of uterine or urinary infections, increased bleeding, constipation, increased hospitalization time, and higher economic costs [80]. The results of a study showed that women who had cesarean section had a greater tendency to do so in later pregnancies and, accordingly, increased their EBF levels compared to their previous one [81].

According to a meta-analysis, the prevalence of cesarean section in Iran was estimated to be 48% [82]. This rate is rather higher when compared to other countries. Since cesarean delivery can have negative effects on the mother and the baby, such as EBF reduction, health policy- and decision-makers in Iran should make a lot of effort to reduce the use of cesarean delivery [83]. In the Health System Transformation Plan (HSTP), which began in 2014 in Iran, much attention has been paid to reducing cesarean delivery in Iran, and health-care service providers have paid for maternity welfare costs to mothers to reduce this kind of delivery [84].

In the present study, the prevalence of EBF in mothers with male children was higher compared to mother with female infants and this is consistent with the results of a study conducted in Saudi Arabia [78] and the findings of study performed in Ghana [85].

Based on the results of our study, the prevalence of EBF in infants whose birth weight was less than 2500 g was higher than that of infants above this weight. Infants with low birth weight are at risk for certain diseases. Breast milk can improve the function of the digestive system, reducing infections [86]. Studies have shown that EBF is a necessity for infants weighing less than 2500 g and should be taken seriously by mothers [87, 88].

Health policy- and decision-makers in Iran have always emphasized the importance and the benefits of EBF and, given its religious, social and economic implications, have implemented broad programs for education and promotion at the community level. Appropriate laws have been approved to promote EBF and supporting mothers during lactation in recent years. In 2011, the maternity leave law was approved for mothers who breastfeed their babies, and according to that, employers can extend maternity leave for a period of 9 months, and the period of maternity leave for triple or more childbirths (1 year receiving salaries). Also, these mothers can come to work 1 h later or leave the workplace 1 h earlier. All government agencies are required to provide female employees with appropriate facilities enabling EBF at the workplace. This law focuses on protecting working women and ensuring their job security, taking into account the specific circumstances of women in lactation, as well as on improving the condition of the growth of their infants.

### Strengths and limitations

Comprehensive search of various scholarly databases, sub-group analysis, meta-regressions and sensitivity analysis were among the strengths of this systematic review and meta-analysis. However, this study also had some limitations, which should be properly recognized. In some Iranian studies, there was no study on the prevalence of EBF. Methodological differences in studies may have led to a high, statistically significant heterogeneity. Low sample size of many studies (21 studies with a sample size less than 500) represents another limitation of the present investigation.

## Conclusion

Our findings indicate that EBF prevalence in Iran was 53%. Undoubtedly, the use of breast milk has many benefits for the baby, and, as such, policy- and decision-makers in the health sector should try to improve maternal care by improving care during pregnancy and after childbirth, giving more education to their mother and their families.

## Supplementary information

**Additional file 1.** Search strategy in PubMed/MEDLINE, Scopus, ISI/Web of Science and Embase databases. Description of data: Details of the search strategy.

## Data Availability

Not applicable.

## Abbreviations

AAP: American Academy of Pediatrics
CI: Confidence Interval
EBF: Exclusive breastfeeding
EMRO: Eastern Mediterranean Regional Office
OR: Odds Ratio
PRISMA: Preferred Reporting Items for Systematic Reviews and Meta-Analyzes
SID: Scientific Information Database
UNICEF: United Nations Children’s Fund
WHO: World Health Organization
