# Supplementary material for: Prevalence of exclusive breastfeeding practice in the first six months of life and its determinants in Iran: a systematic review and meta-analysis
Source: BMC Pediatr. 2019 Oct 27;19:384. doi: 10.1186/s12887-019-1776-0 (PMC6815441; doi:10.1186/s12887-019-1776-0)
Supplement: Supplementary file 1 — Additional file 1. Search strategy in PubMed/MEDLINE, Scopus, ISI/Web of Science and Embase databases. Description of data: Details of the search strategy. [file 12887_2019_1776_MOESM1_ESM.docx]

**PubMed search strategy:**

#1 exclusive breastfeeding[Ti/ab]

#2 breastfeeding[Ti/ab]

#3 breast-feeding[Ti/ab]

#4 breastfeeding patterns[Ti/ab]

#5 breastfeeding practices[Ti/ab]

#6 breastfeeding status[Ti/ab]

#7 feeding status[Ti/ab]

#8 #1 OR #2 OR #3 OR #4 OR #5 OR #6 OR #7

#9 frequency[Ti/ab]

#10 epidemiology[Ti/ab]

#11 prevalence[Ti/ab]

#12 patterns[Ti/ab]

#13 assessment[Ti/ab]

#14 investigation[Ti/ab]

#15 #9 OR #10 OR #11 OR #12 OR #13 OR 14

#16 Iran[Ti/ab]

#17 #8 AND #15 AND #16

**Scopus search strategy:**

#1 exclusive breastfeeding[Tile/abstract]

#2 breastfeeding[Tile/abstract]

#3 breast-feeding[Tile/abstract]

#4 breastfeeding patterns[Tile/abstract]

#5 breastfeeding practices[Tile/abstract]

#6 breastfeeding status[Tile/abstract]

#7 feeding status[Tile/abstract]

#8 #1 OR #2 OR #3 OR #4 OR #5 OR #6 OR #7

#9 frequency[Tile/abstract]

#10 epidemiology[Tile/abstract]

#11 prevalence[Tile/abstract]

#12 patterns[Tile/abstract]

#13 assessment[Tile/abstract]

#14 investigation[Tile/abstract]

#15 #9 OR #10 OR #11 OR #12 OR #13 OR 14

#16 Iran[Tile/abstract]

#17 #8 AND #15 AND #16

**ISI/Web of Science search strategy:**

#1 TS= (exclusive breastfeeding* OR breastfeeding* OR breast-feeding* OR breastfeeding patterns* OR breastfeeding practices* OR breastfeeding status* OR feeding status)

#2 TS= (frequency* OR epidemiology*OR prevalence* OR patterns*OR assessment* OR investigation)

#3 TS= (Iran)

#4 #1 AND #2 AND #3

**Emabe search strategy:**

#1 ‘exclusive breastfeeding’: ab,ti

#2 ‘breastfeeding’: ab,ti

#3 ‘breast-feeding’: ab,ti

#4 ‘breastfeeding patterns’: ab,ti

#5 ‘breastfeeding practices’: ab,ti

#6 ‘breastfeeding status’: ab,ti

#7 ‘feeding status’: ab,ti

#8 #1 OR #2 OR #3 OR #4 OR #5 OR #6 OR #7

#9 ‘frequency’: ab,ti

#10 ‘epidemiology’: ab,ti

#11 ‘prevalence’: ab,ti

#12 ‘patterns’: ab,ti

#13 ‘assessment’: ab,ti

#14 ‘investigation’: ab,ti

#15 #9 OR #10 OR #11 OR #12 OR #13 OR 14

#16 ‘Iran’: ab,ti

#17 #8 AND #15 AND #16
